# Robot Pose Estimation and Normal Trajectory Generation on Curved Surface Using an Enhanced Non-Contact Approach

**DOI:** 10.3390/s23083816

**Published:** 2023-04-07

**Authors:** Syed Humayoon Shah, Chyi-Yeu Lin, Chi-Cuong Tran, Anton Royanto Ahmad

**Affiliations:** 1 Department of Mechanical Engineering, National Taiwan University of Science and Technology, Taipei 106, Taiwan; 2 Center for Cyber-Physical System, National Taiwan University of Science and Technology, Taipei 106, Taiwan; 3 Taiwan Building Technology Center, National Taiwan University of Science and Technology, Taipei 106, Taiwan; 4 Department of Mechanical Engineering, Universitas Ibn Khaldun Bogor, Bogor 16162, Indonesia

**Keywords:** pose correction, robot surface tracking, RGB-D camera

## Abstract

The use of robots for machining operations has become very popular in the last few decades. However, the challenge of the robotic-based machining process, such as surface finishing on curved surfaces, still persists. Prior studies (non-contact- and contact-based) have their own limitations, such as fixture error and surface friction. To cope with these challenges, this study proposes an advanced technique for path correction and normal trajectory generation while tracking a curved workpiece’s surface. Initially, a key-point selection approach is used to estimate a reference workpiece’s coordinates using a depth measuring tool. This approach overcomes the fixture errors and enables the robot to track the desired path, i.e., where the surface normal trajectory is needed. Subsequently, this study employs an attached RGB-D camera on the end-effector of the robot for determining the depth and angle between the robot and the contact surface, which nullifies surface friction issues. The point cloud information of the contact surface is employed by the pose correction algorithm to guarantee the robot’s perpendicularity and constant contact with the surface. The efficiency of the proposed technique is analyzed by carrying out several experimental trials using a 6 DOF robot manipulator. The results reveal a better normal trajectory generation than previous state-of-the-art research, with an average angle and depth error of 1.8 degrees and 0.4 mm.

## 1. Introduction

Over the last few decades, the demand for workpiece surface finishing has increased in different industries, such as machinery, automotive, aerospace, and others, as it is one of the salient operations that need to be carried out to improve the quality of a product. Traditionally, surface finishing operations have been conducted predominantly through skilled workers, which results in inconsistencies in the finishing quality of the product, is inefficient, and poses health risks to the workers [1,2,3,4]. Industrial robots (6 degrees of freedom) have the ability to significantly improve and automate the surface finishing process [5], considering their flexibility, mechanical reconfigurability, and reliability as compared to other approaches [6,7,8,9]. Despite the high utilization of manipulator robots in industrial applications, such as palletizing and depalletizing, their usage is still very low in complex machining operations, such as surface finishing, due to challenges such as complex programming, poor accuracy, and insufficient rigidity [10].

The development of an appropriate trajectory for a surface finishing tool is perhaps one of the key aspects of an automatic machining operation. In a surface finishing operation, the shape of the path plays a significant role in the smoothness and uniformity of the workpiece’s surface. The literature documents three main classifications of workpiece contour acquisition methods, including 3D Scan/CAD data, surface tracking techniques using force sensors, and image processing. Wang et al. [11] used the workpiece’s CAD data to realize the actual shape of surfaces. Another commonly used approach for acquiring surface paths is 3D scanning; however, this approach ignores fixture uncertainty, and it is typically challenging to match the part’s surface to that of the CAD model because the aforementioned approach does not account for the part’s orientation. On the other hand, a contact-based method that estimates normal contact with the surface by utilizing the feedback information from a force/torque sensor has been documented in the literature [11,12,13,14]. The obtained force information is fed back into the algorithm to estimate the desired angle for acquiring a perpendicular relationship between the robot’s end-effector and the contact surface. However, these systems are prone to errors due to surface friction and the characteristics of the workpiece material. Moreover, for retrieving depth information, the hybrid vision and force servoing approaches are presented, which are capable of generating 3D Cartesian position coordinates for controlling the robots (refer to [15,16,17]). These techniques cannot be utilized to construct robot trajectories with precise tool orientation control because pitch and yaw angles are not regulated. Conversely, robotic tooling tasks, such as polishing, need constant contact between the robot and the contour surface. To realize satisfactory surface finishing, the contact force is required to be controlled properly. Maintaining constant contact between the robot’s end-effector and the contact surface has been addressed by several approaches, such as force control, position control, or a combination of both, as can be seen in [18,19,20,21,22,23,24].

Studies in the literature have attempted to address these challenges using the aforementioned techniques. However, several issues still exist, such as low accuracy, surface friction, and fixture error. This study proposes a stereo camera-based approach to construct a normal trajectory by tracking the surface of the object. In addition, a depth measurement tool is employed to make sure that the robot tracks the desired path. Concretely, the main contributions of this study can be summed up as follows:

(1) A depth measurement tool is employed with a key-point selection approach to address the challenge of absolute error. For instance, the robot needs to track the desired path while generating the normal trajectory.

(2) An RGB-D camera is used to estimate the contact state between the workpiece’s surface and the robot’s end-effector. The camera collects point cloud information of the target surface. The point cloud information is employed to estimate the surface normal on the surface of the curved object. This allows for correcting the robot’s pose by maintaining a target normal to the surface.

(3) The performance of the presented technique is experimentally tested on a curved surface with a 6 DOF manipulator using a Cartesian pose controller to follow the predetermined route and construct a new normal trajectory for the robotic-based machining operation.

The remainder of the paper is structured as follows: Following the Introduction, the state-of-the-art literature review is presented. Section 2 demonstrates an overview of the proposed system. Section 3 describes the robot trajectory correction algorithm. Section 4 discusses the calibration techniques utilized in this study. Section 5 describes the experimental work and the results obtained. Finally, Section 6 presents the conclusion of the research work completed for this article.

### State-of-the-Art Literature

This section presents the state-of-the-art literature review of the proposed scheme for robot normal trajectory generation.

A deformation in the surface of a workpiece may arise as a result of casting or clamping and gravity forces, which are significant to consider for machining applications using industrial robots, such as robotic surface finishing. To overcome this issue, one of the extensively documented approaches in the literature is to realize the transformation between the measured 3D model using sensors and the actual part. This can be obtained by analyzing a CAD model’s point cloud information against the data provided by a 3D measurement instrument [25]. By employing the registration algorithm, a transformation between the two point clouds is estimated. For better performance, initially, an approximate alignment must be estimated if the point clouds are not aligned. The final transformation can be measured with the help of a registration algorithm that can be used to adjust the trajectory of a reference robot. Similar research has suggested adjusting the tool path based on a CAD model and using direct teaching methods with a matching algorithm [26]. Additionally, their study incorporated impedance control and a virtual wall technique to avoid excessive contact force and to enhance the performance of the force control algorithm.

Likewise, H. Kosler et al. [27] proposed an adaptive system to address the position and orientation errors that occur during deburring of a workpiece. Their study employed a custom-built laser-triangulation sensor to identify the 3D structure of the surface of the workpiece. In addition, the research utilized the ICP registration algorithm to estimate the orientation and position deviation with respect to the reference model, which is determined by applying robot teaching to a reference workpiece that has already been deburred. The current processed part’s errors are adjusted by rotating and translating the tool path in accordance with the registration findings. The performance of the presented adaptive technique is experimentally validated.

Similarly, Kuss et al. [28] described a method for measuring workpiece shape irregularities and adjusting the tool path in a robotic deburring system. The product design provided dimensional tolerance information for manufactured parts, which was utilized to produce variants of the CAD model and suitable reference point clouds of the workpiece. The authors further employed a matching technique based on an iterative closest point algorithm between the reference cloud and the point cloud obtained from the manufactured part. The structural resemblance of analyzed point clouds was determined by comparing the distances between the respective point clouds. Eventually, for subsequent trajectory planning and workpiece mapping, the geometrical framework with the best matching geometry was adopted. The performance of the presented system was evaluated by a virtual test case and employing a stereo camera (for shape sensing) mounted on an industrial robot.

Recently, Amersdorfer et al. [29] proposed a three-laser distance sensor positioned around the polishing tool for estimating the surface normal of the polishing contact surface. However, this technique falls short when dealing with unknown uneven surfaces since it cannot determine the polishing pad’s current state of contact with the surface. To overcome this issue, Wang et al. [30] employed a three-laser distance sensor and a linear encoder. The experimental results of their study showcase a 3.33 degree average error.

Conversely, Lin et al. [31] suggested a contact-based method recently for contact state estimation. The authors employed a multi-axis force/torque sensor with a self-developed tool. The force sensor feeds back the force information when the tool makes contact with the workpiece’s surface. The algorithm running on the computer estimates the modification needed for the robot to make normal contact with the surface. However, due to the surface friction issue, the scheme still required considerable development to be used effectively. For the sake of simplification, a summary of the recently documented literature is given in Table 1.

## 2. System Overview

In this work, a scheme is proposed and developed for coping with the kinematic uncertainties and complex surface trajectory tracking challenges. The proposed scheme addresses two main challenges involved in normal trajectory generation on complex surfaces, i.e., tracking the desired path on which the normal trajectory is required to be generated and then generating a normal trajectory on that desired path. For tracking the desired path, a key-point selection approach is employed by using a depth measurement tool (DMT). The DMT is positioned on the robot’s flange with the help of a 3D-printed holder. The DMT is moved to three different corners of the workpiece with similar depths and different x- and y-axes at each corner for calibrating the robot to the workpiece. In this way, the origin of the workpiece’s coordinate frame is determined, which aids in tracking the desired path.

**Figure 1:**
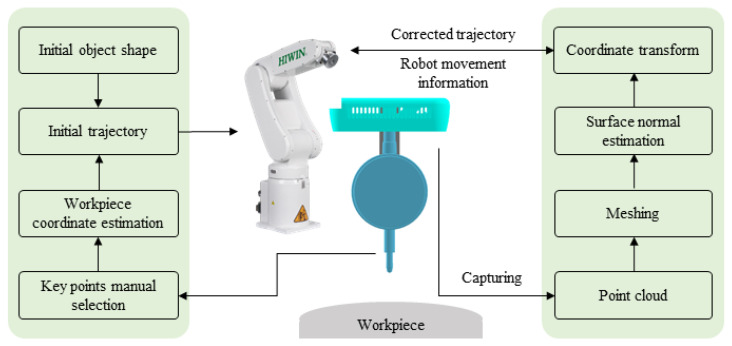
Proposed methodology for desired path tracking and surface normal trajectory generation.

For the generation of the normal trajectory, a depth camera is used to calculate normal to the surface while tracking the desired path. The camera is mounted on the robot flange to obtain the point cloud information from the workpiece’s surface. The point cloud information is used to determine the angle and depth for the robot’s end-effector to realize normal contact with the surface. In this way, a normal trajectory, i.e., a trajectory in which the tool is perpendicular to the surface, is generated on the desired path. The acquired trajectory ensures normal contact of the tool with the curved surface and can be utilized in machining operations on a complex surface with the robot. The overall framework of the presented scheme is illustrated in Figure 1.

**Figure 2:**
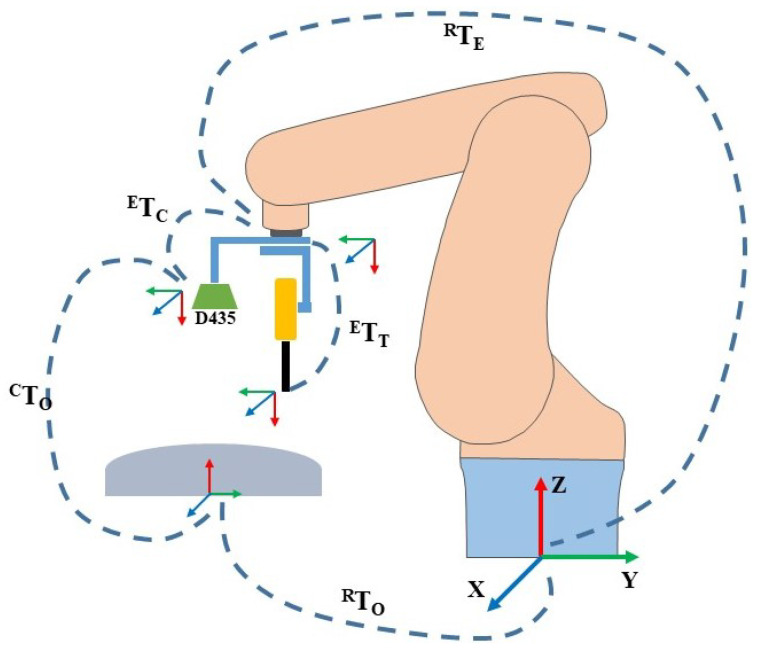
Overall transformation matrices used in the proposed scheme. Where the term ${}^{R}T_{O}$ is object-to-robot transformation.${}^{R}T_{E}$ represents the transformation from the robot to the end-effector, ${}^{E}T_{C}$ is the transformation from the end-effector to the camera, and ${}^{C}T_{O}$ is camera-to-workpiece transformations. ${}^{E}T_{C}$ sets the camera’s relative position and rotation in relation to the robot’s pose, and ${}^{R}T_{E}$ is the current position of the end-effector relative to the robot’s base coordinate. RealSense D435 is the stero camera employed.

For practical use of the system, a transformation matrix between the camera coordinate and robot flange coordinate must be computed. A transformation matrix integrates both the rotation matrix and the translation vector into one 4 × 4 matrix, as given in the following equation.

(1) $$T=\left[\begin{array}{cc} R & P\\ 0 & 1 \end{array}\right]$$

where the term *R* represents a 3×3 rotation matrix, and *P* = $\left(t_{1},t_{2},t_{3}\right)$ denotes a 3×1 translation/position vector. These are the extrinsic parameters of the camera and depth measuring tool, which are derived using the calibration process and explained in detail in Section 4. Moreover, another transformation matrix is required between the depth measurement tool and the robot flange. Figure 2 depicts the relationship between all the transformation matrices that must be estimated. The final objective is to compute the object-to-robot transformation ${}^{R}T_{O}$, which is unknown, by finding the relationships between other coordinate frames. Similarly, the term ${}^{R}T_{E}$, in Figure 2, represents the transformation from the robot to the end-effector. In addition, ${}^{E}T_{C}$ represents the transformation from the end-effector to the camera, which is estimated with the help of a hand–eye calibration technique, and ${}^{C}T_{O}$, which is also unknown, represents camera-to-workpiece transformations. The overall equation is given as ${}^{R}T_{O}$ = ${}^{R}T_{E}$${}^{E}T_{C}$${}^{C}T_{O}$. In other words, ${}^{E}T_{C}$ sets the camera’s relative position and rotation in relation to the robot’s pose, and ${}^{R}T_{E}$ is the current position of the end-effector relative to the robot’s base coordinate. A calibration process known as hand–eye calibration is used to obtain this camera-to-robot transformation and is discussed in detail in Section 4.1.

## 3. Robot Trajectory Correction Algorithm

**Figure 3:**
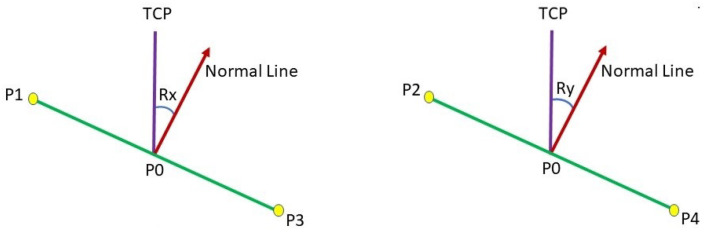
The deflection angle from TCP to normal line.

It is essential to know the contact state of the tool and workpiece in order to make the tool normal to the contact surface and maintain a constant depth throughout the surface [32]. It needs to estimate the deflection angle (ΔRx and ΔRy between the current and the normal (desired) contact state, as demonstrated in Figure 3.

To address this problem of determining the surface normal on unknown surfaces, there are primarily two approaches documented in the literature, i.e., contact-based and non-contact-based. The contact-based approach uses sensors, such as force sensors, and dedicated contact tools [31]. This study deals with a novel approach using a stereo camera and a depth measurement tool. The aim is to improve the measurement accuracy of the normal trajectory generation approaches. The proposed method makes use of data from a point cloud for approximating the angle must be altered on the surface to establish normal surface contact.

The system can examine the various joint configurations and positions along the path using the robot’s inverse kinematics and object coordinates, delivering information regarding singularities and out-of-workspace conditions. We encountered the following mathematical challenge when estimating the motion parameters of a rigid object using 3D point correspondences, which involves figuring out the relative attitude of a rigid object’s set points *A* = $a_{1},a_{2},\ldots ,a_{n}$ with regard to a reference’s set points *B* = $b_{1},b_{2},\ldots ,b_{n}$.

(2) A=X×B

with

(3) $$X=\left[\begin{array}{cc} R & t\\ 0 & 1\; \end{array}\right]=SE\left(R,t\right)\epsilon SE\left(3\right)$$

where *X* is the unknown, *R* represents the rotation, and *t* is the translation. The equation can be expressed in the following manner:

(4) $$a_{i}=Rb_{i}+t$$

Our objective is to minimize *E* by obtaining *R* and *t*:

(5) $$E=\sum_{i_{1}}^{N}{∥a_{1}-(Rb_{i}+t)∥}^{2}$$

To emphasize the determination of the rotation *R*, we can reformulate the problem by enforcing the condition that the translation is equal to zero:

(6) $$x_{i}\overset{\Delta }{=}a_{i}-a$$

(7) $$y_{i}\overset{\Delta }{=}b_{i}-b$$

(8) $$E=\sum_{i_{1}}^{N}{∥x_{1}-Ry_{i}∥}^{2}$$

Minimizing *E* is equivalent to maximizing:

(9) $$F=\sum_{i=1}^{N}x{}_{i}{}^{T}Ry_{i}=Tr\left(\sum_{i=1}^{N}Ry_{i}x{}_{i}{}^{T}\right)=Tr\left(RH\right)$$

where

(10) $$H\overset{\Delta }{=}\sum_{i=1}^{N}x_{i}y_{i}{}^{T}$$

Through the utilization of the SVD method, we are able to effectively and accurately obtain the covariance matrix *H*:

(11) $$H=U\times V{}^{T}$$

Translation can be determined by using

(12) t=A−RB

The initial path on the workpiece’s surface is provided to the robot to track using the key-point selection method. This path is used as a reference trajectory (also referred to as the desired path) for subsequent normal trajectory generation tasks. The tool center point (TCP) is set 100 mm from the surface of the workpiece in order to calculate the target point on the surface with respect to the camera. During path tracking, the robot moves along the x-coordinates while maintaining the constant set distance. From the point cloud information, Equations (13) and (14) measure the angle adjustment required for obtaining normal contact between the robot and the target point of the trajectory. This procedure is repeated until the robot successfully follows the entire desired path throughout the surface of the workpiece. Consequently, a normal trajectory is developed by regulating the depth and posture of the robot relative to the surface while tracking the surface. The generated trajectory ensures normal contact of the robot with the surface of the curved workpiece.

**Figure 4:**
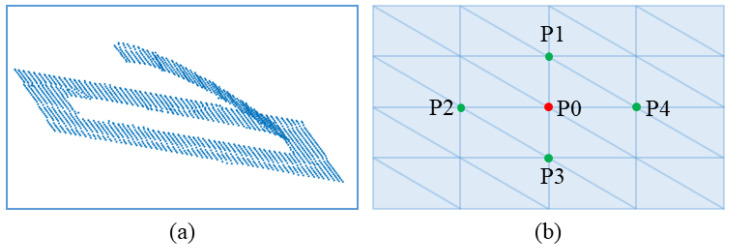
Demonstrate 5 points representation. (**a**) Point cloud model of the workpiece, (**b**) A set of five extracted points.

Generating a normal on a single point through the stereo camera involves the collection of appropriate comparable points in the 3D point cloud based on the target point (*P*0) and the selection of four key-points, i.e., *P*1, *P*2, *P*3, and *P*4. Five points were created, as showcased in Figure 4. These points were utilized to estimate the surface normal angles $\Delta R_{x}$ and $\Delta R_{x}$ on the contact surface for the robot, as demonstrated in Figure 3.

In Figure 3, the Cartesian coordinate information of *P*1, *P*2, *P*3, and *P*4 are employed to estimate the surface normal at the target point (*P*0) via Equations (13) and (14).

(13) $$\Delta R_{x}=\tan^{-1}\frac{P1_{z}-P3_{z}}{\sqrt{{\left(P1_{x}-P3_{x}\right)}^{2}+{\left(P1_{y}-P3_{y}\right)}^{2}}}$$

(14) $$\Delta R_{y}=\tan^{-1}\frac{P2_{z}-P4_{z}}{\sqrt{{\left(P2_{x}-P4_{x}\right)}^{2}+{\left(P2_{y}-P4_{y}\right)}^{2}}}$$

Unlike conventional cameras, which need complex calculations to estimate the depth information from the camera to the object, our proposed approach uses a stereo camera, which provides 3D information (*x*, *y*, *z*) of the workpiece’s surface, making it easy to estimate the surface normal of the contact surface.

## 4. Calibration Methods

This section presents the essential calibration approaches (such as hand–eye, depth measurement tool, and robot) needed prior to performing experiments. These calibration techniques are discussed in detail in the follow-up subsections.

### 4.1. Hand-Eye Calibration

Hand-eye calibration plays a vital role in vision-based control. The process of estimating the geometric synchronization among a robot’s end-effector, a sensor (i.e., camera), and the environment is known as hand-eye calibration [33] in the context of robotics. In other words, it calculates the transformation matrix (rotation and translation) of the robot flange and camera by constructing a mathematical model between the coordinates of the camera and the robot flange. The camera is rigidly coupled to the robot gripper, and the checkerboard is fixed to one position in this kind of calibration. The manipulator needs to have adequate DOF to allow the camera to move around two distinct axes while keeping the camera focused on a checkerboard to determine the 3D mathematical relation between the camera and the robot flange.

A plethora of literature has been documented on different approaches evolved over the years for hand–eye calibration; interested readers can refer to [34]. To the best of our knowledge, the conventional checkerboard method is the most prevalent and well-established methodology for hand-eye calibration, particularly for an industrial robot manipulator with a camera attached to its flange.

**Figure 5:**
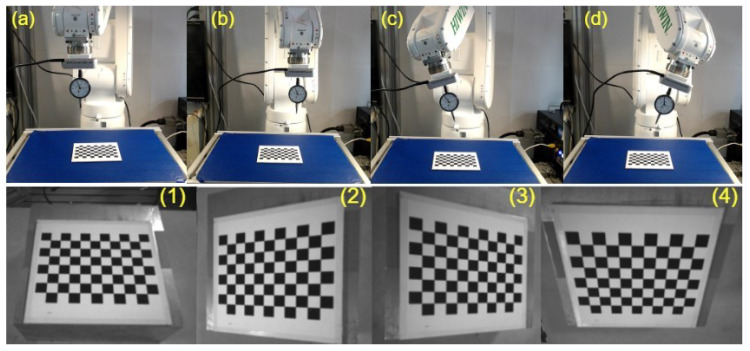
Hand-eye calibration process utilized. (**a**–**d**) Shows robot different poses, (**1**–**4**) Showcases the corresponding images captured during the robot’s execution of poses (**a**–**d**).

Thus, this study used the conventional hand–eye calibration method, as shown in Figure 5. Considering the FOV of the camera utilized, a 7×7 mm checkerboard was placed on the working table. The camera mounted on the flange was then rotated around the x- and y-axes of the robot’s end-effector at several random angles. The images obtained with the aforementioned process are employed to estimate the final homogeneous transformation matrix between the camera and the robot’s flange using the approach developed by Tasi et al. [35].

### 4.2. Depth Measurement Tool Calibration

**Figure 6:**
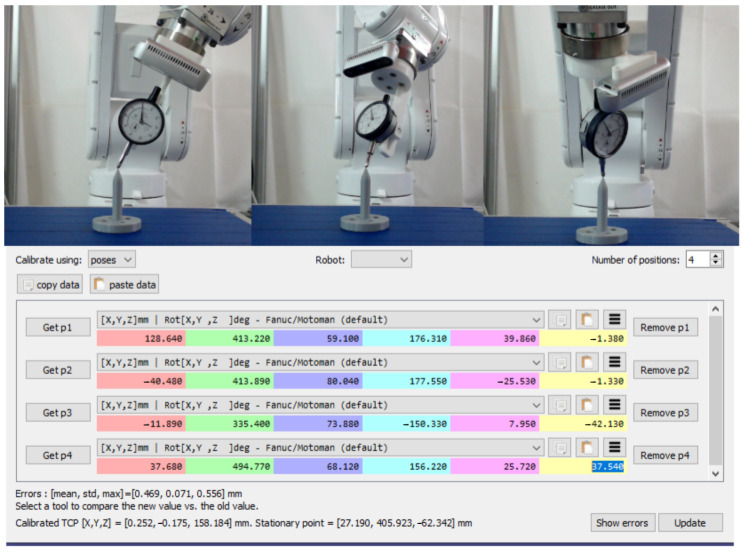
Calibration method of the depth measurement tool.

The robot merely returns the coordinates of its flange. However, we may approach the contact surface from the current center point of the depth measurement tool. Thus, it is necessary to compute the offset between the robot’s flange and the center point of the mounted tool (DMT). To obtain the TCP, the procedure calibrate-robot-touching-point is utilized which is available in RoboDK software, as shown in Figure 6. The idea is to touch the same point with the robot’s contact point at least three times while turning the gripper around two axes. Multiple methods can be employed to communicate with the robot to reach the point and rotate along some axes. In this study, a manual approach is used to contact the 3D-printed touching point and manually type the robot coordinate values in the RoboDK software. After touching the same spot a couple of times with different robot orientations, the software predicts the offset between the robot flange and the TCP based on the robot positions, as shown in Figure 6. In order words, the robot’s movements involve both translation and rotations to minimize the error between the robot’s flange and the TCP. The acquired information can be grouped together to form an equation of the form *AX* = *XB*. By minimizing this type of equation, it is possible to determine the position of the supporting frame in relation to the robot flange. This calibration procedure has from a 0.2 to 0.3 mm inaccuracy for a typical uncalibrated robot [36]. Due to the fact that we only captured four alternative robot orientations, the inaccuracy in our case is 0.46 mm. By gathering robot coordinate information for numerous orientations, this inaccuracy can be minimized.

### 4.3. Robot Calibration

The robot must calibrate to the workpiece in order to estimate its reference coordinate frame. We employed the calibration feature of the robot teaching pendant to carry out this calibration. Utilizing the three key-point selection approach, the reference coordinates for the workpiece were acquired by using a depth measurement tool mounted on the robot flange. The three-point selection method touches the working table near the workpiece at three different locations using the TCP (DMT) and obtains the position and orientation information of the robot’s end-effector at each position. The teaching pendant of the HIWIN robot has a robot calibration feature that allows the insertion of the robot’s pose information, which is manually obtained by moving the robot into three distinct poses in the directions of the x- and y-axes. At each location, the depth reading on the depth measurement tool is fairly equivalent. A reference frame for the workpiece is established using the aforementioned information. This reference coordinate of the workpiece is used to track the desired path on the workpiece’s surface CAD in order to obtain the normal trajectory at the end of the surface tracking operation.

## 5. Experimental Results

This section uses an experimental robot validation to demonstrate the viability of the presented techniques for use with robotic manipulators on curved surfaces.

### 5.1. Experimental Setup

**Figure 7:**
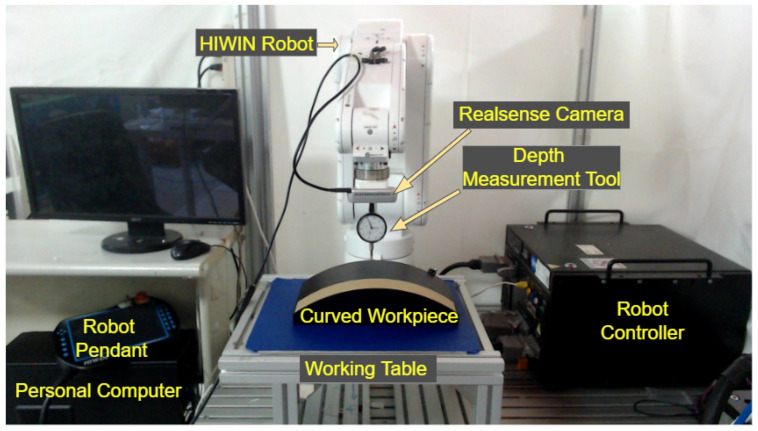
The experimental hardware used to evaluate the efficacy of the presented scheme.

The performance of the presented strategy is validated by utilizing the experimental hardware shown in Figure 7.

The experimental setup consists of a 6 DOF articulated HIWIN (Taichung City, Taiwan) robot that is controlled by a motion controller unit. In addition, a RealSense stereo camera and depth measurement tool are coupled together and mounted on the robot’s flange using a customized 3D-printed holder. A master computer with a GPU RTX-1060 running the C++ programming language, a working table, and a curved workpiece are also part of the experimental hardware.

The six-axis manipulator is connected to the controller unit via serial communication. Additionally, the TCP/IP protocol is used to transmit and receive data between a personal computer and the robot controller unit. For instance, the robot controller receives the updated Cartesian position and orientation of the robot from the computer via Ethernet. The controller unit estimates the joint angles using inverse kinematics for the particular pose. The joint angles are transmitted to the joint motors of the manipulator through serial communication, to assist the end-effector of the robot to reach that particular pose. Similarly, the stereo camera (RealSense D435 manufactured by Intel Corporation, Santa Clara, CA, USA). is utilized to acquire the point cloud data of the target surface, and a USB connection is established to transport data from the stereo camera to the computer. The used RealSense camera is capable of producing high-quality point cloud data at a reasonable resolution and better performance, accuracy, and frame rate. Additionally, it can follow movements at speeds of up to 40 m/s by capturing high-density point clouds at a frame rate that is almost in real time. Furthermore, it has an RGB sensor with a global shutter and an 86-degree field of view. To communicate between the camera and the computer, serial communication is employed.

### 5.2. Normal Trajectory and Constant Contact Experiments

This study proposes a method to track the desired path and generate a surface normal trajectory with constant surface contact on a multidimensional geometric surface, leveraging data from a depth measurement tool coupled with a stereo camera. The efficacy of the proposed scheme is analyzed on three different workpiece surfaces, including (1) a flat surface made up of steel, (2) a customized surface designed with different degree offsets (such as 0, 10, 15, 18, 20) in solid and 3D-printed works, and (3) a curved surface. The curved workpiece is machined specifically for this study to compare the obtained normal trajectory based on the proposed scheme with the estimated CAD model trajectory (referred to as the desired trajectory). The maximum height of the curved workpiece is 30 mm, the width is 160 mm, and the total length is 300 mm. The robot moves at a constant speed and scans the surface through a camera mounted on the flange obtaining point cloud information (PCI) of the contact surface. The Cartesian coordinate information of the point cloud of the target point is used to estimate the depth and angle corrections required to obtain the surface normal and maintain contact with the curved surface using the surface normal correction Equations (13) and (14).

**Figure 8:**
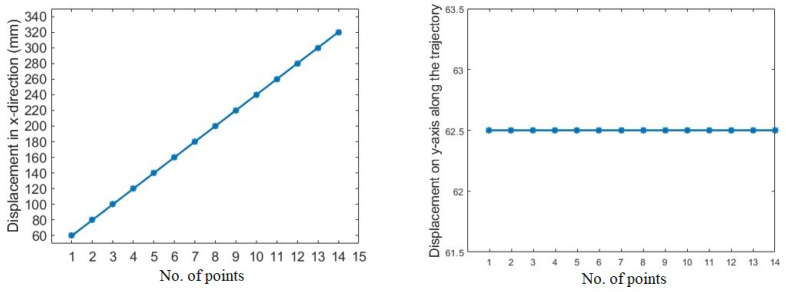
Movement of the robot along x- and y-axes.

**Figure 9:**
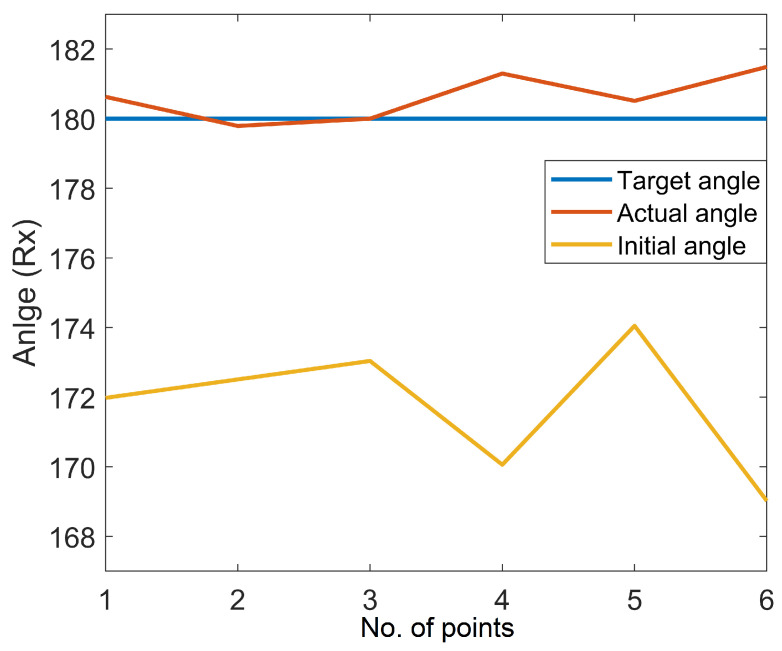
Experimental result on the flat surface.

**Figure 10:**
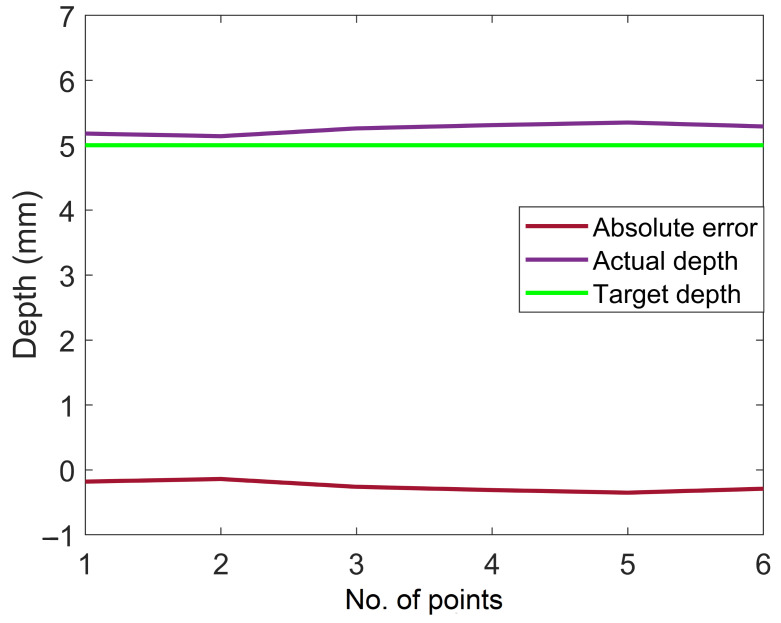
Absolute depth error in the normal trajectory generation on the flat surface.

As mentioned earlier, three different scenarios are utilized to assess the system’s performance. Initially, experiments were carried out on the surface of a flat workpiece to analyze the efficiency of the proposed scheme in rectifying deviated angles and to obtain a constant depth (z-axis). This case is a simple and handy tool to evaluate the performance of the system due to a known surface normal angle, i.e., 180 degrees (Rx) when the robot makes normal contact with the flat surface. The speed of the robot is constant and moves along the x-axis. In addition, the y- and z-axes are unchanged during tracking the path on the flat surface, as shown in Figure 8. The acquired angle correction results after surface tracking are demonstrated in Figure 9. Figure 9 showcases the initial, actual, and target angles of Rx (α) at each point along the trajectory. The vertical axis of the graph shows the angles in degrees, and the horizontal axis of the graph illustrates the number of points along the desired path. The average maximum angle absolute error in the Rx angle is 1.0 degrees, and the average minimum absolute error is 0.3 degrees. Moreover, Figure 10 illustrates the absolute error (actual target) obtained during the correction of the depth along the even surface trajectory. The average maximum absolute error in depth is 0.3 mm, and the average minimum absolute error is 0.1 mm.

**Figure 11:**
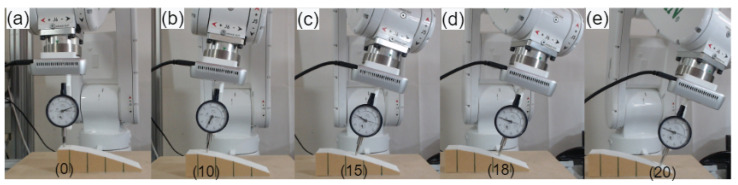
Single point normal estimation on the customized workpiece. (**a**–**e**) Shows correction of robot angles on customized workpiece with varying angle offsets (0°, 10°, 15°, 18°, 20°).

Similarly, multiple tests were performed on a customized 3D-printed workpiece’s surface for angle correction validation, as illustrated in Figure 11.

The workpiece was created with several known surface angles in SolidWorks software before being 3D printed. For instance, the top surface has zero angle deviation, whereas the other four surfaces have different angle deviations from the normal (flat surface). The other subsequent surfaces have angle offsets of 10, 15, 18, and 20 degrees. Additionally, a target point at the center of the surfaces was defined to determine angle deviation at that particular point. The movement of the robot was in the x-direction with a required operating distance of the camera from the workpiece’s surface. The camera collects the point cloud information of the desired point on the surface and estimates the angle adjustment needed for each surface based on Equations (13) and (14). The depth measuring tool was used to make contact with each surface of the workpiece after rectification in order to ensure tool contact with the surface and compare the angle correction with the angle offset of the surface. For instance, the robot must tilt from 180 degrees to 170 degrees on a surface with a 10-degree angle deviation in order to make perpendicular contact with the surface. The average minimum angle error on these five distinct surfaces was 0.3 degrees, and the average maximum angle error was 1.3 degrees. The experimental findings showcase the effectiveness of the proposed method.

**Figure 12:**
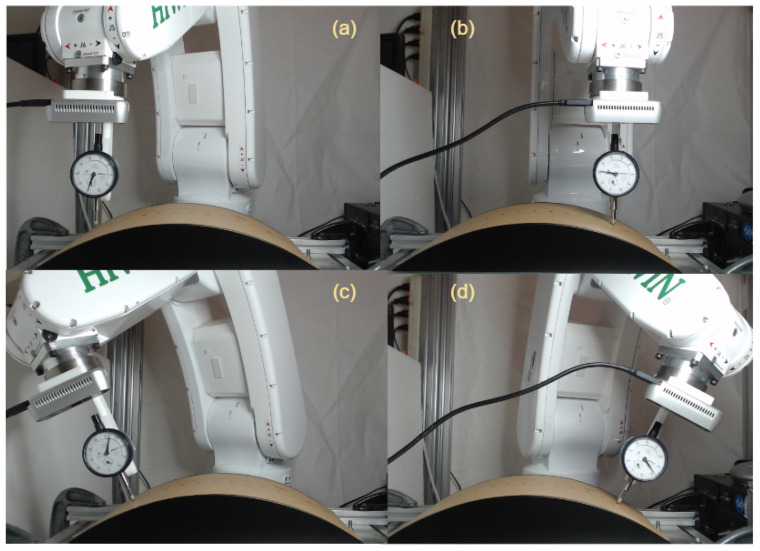
Experiments on curved surface. (**a**,**b**) Demonstrate incorrect robot poses where the end-effector is not perpendicular to the surface, while (**c**,**d**) Showcase the corrected poses where the end-effector is perpendicular to the surface.

Finally, normal surface trajectory generation experiments were then conducted on the curved surface, as demonstrated in Figure 12. A total of 15 points (not perpendicular to the surface) were selected on the curved surface to generate a normal trajectory. The robot moves along the surface trajectory at a constant speed with an adjustable camera working distance positioned on the robot flange. The robot moves along the x-axis, while the movement along the y-axis is kept constant, and the movement along the z-axis is kept in such a way to ensure the working distance of the stereo camera. Initially, the robot estimates the reference coordinate of the workpiece using a contact-based key-point selection approach. Using the reference coordinate, the robot starts tracking the desired path. The camera senses the points on the desired path and derives the point cloud information on each point while tracking the curved surface. The Cartesian coordinate information of the points is fed back to the algorithm, which estimates the angle adjustment. As a result, a normal trajectory is generated for the curved workpiece’s surface. Figure 12a, b show that the robot is not normal to the surface, i.e., incorrect pose while Figure 12c, d illustrate the corrected pose, i.e., the end-effector is perpendicular to the surface.

**Figure 13:**
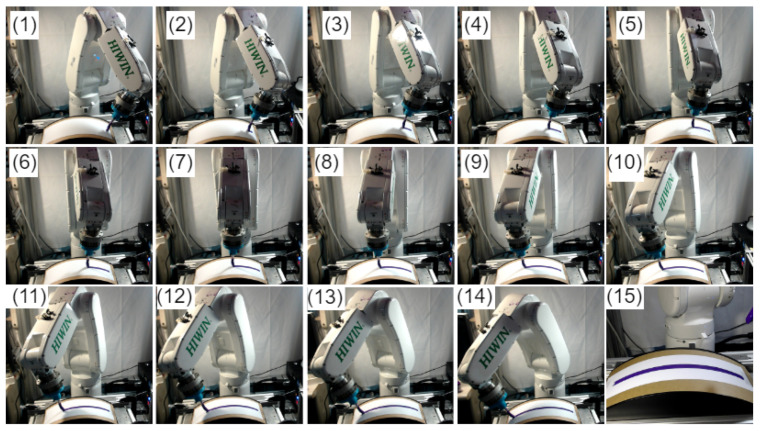
Robot painting a paper employing the obtained normal trajectory: (**1**–**14**) Display the robot while using the normal trajectory to paint the curved surface, while (**15**) depicts the completed painted trajectory.

**Figure 14:**
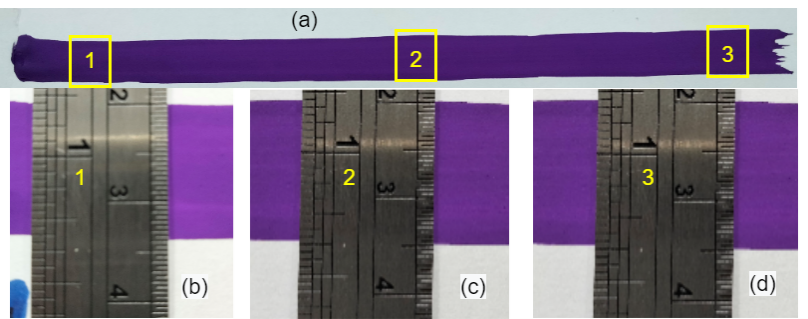
The final results of robot painting: (**a**) Complete painted trajectory; (**b**–**d**) Show the 1, 2, and 3 points of (**a**).

The acquired normal trajectory was validated by painting a white page on the same curved surface for which the normal trajectory is generated, as shown in Figure 13. This technique is one of the possible methods to verify the normal trajectory by measuring the width of the trajectory at different points. For instance, if the overall trajectory is normal, and the contact force is similar at all points, then the width of the painted trajectory should be similar at all points. The length and installation position of the painting brush is similar to that of the depth measurement tool. For example, the length of the tool is 157 mm, so the length of the painting brush is also kept at 157 mm. Figure 13 (1–14) illustrates the robot while using the normal trajectory to paint the curved surface. Figure 13 (15) demonstrates the completed painted trajectory. Finally, the width of the painted trajectory was measured with a ruler on three different points as start, middle, and endpoints, as showcased in Figure 14. The maximum deviation recorded in width was 0.1 mm. This shows that the robot’s end-effector (brush) is perpendicular and has constant contact with the surface, which verifies the significance of this study.

As stated earlier, the curved workpiece is specifically designed for this study, and a CAD model is available. In order to modify the trajectory in both the x- and y-axes, unlike prior studies, a reference path (not normal to the surface) was constructed from the available workpiece’s CAD model. This allows us to analyze the efficiency of the presented scheme in all possible ways. The experiments were conducted while tracking the reference path (using the key-point selection approach) on the curved workpiece’s surface. For surface normal trajectory generation experiments, a total of 32 points were selected from the CAD model trajectory. The robot moves along the surface trajectory at a constant speed with an adjustable camera working distance positioned on the robot flange. The movement of the robot is along the x- and y-axes, and the movement along the z-axis is kept in such a way as to ensure the working distance of the stereo camera.

**Figure 15:**
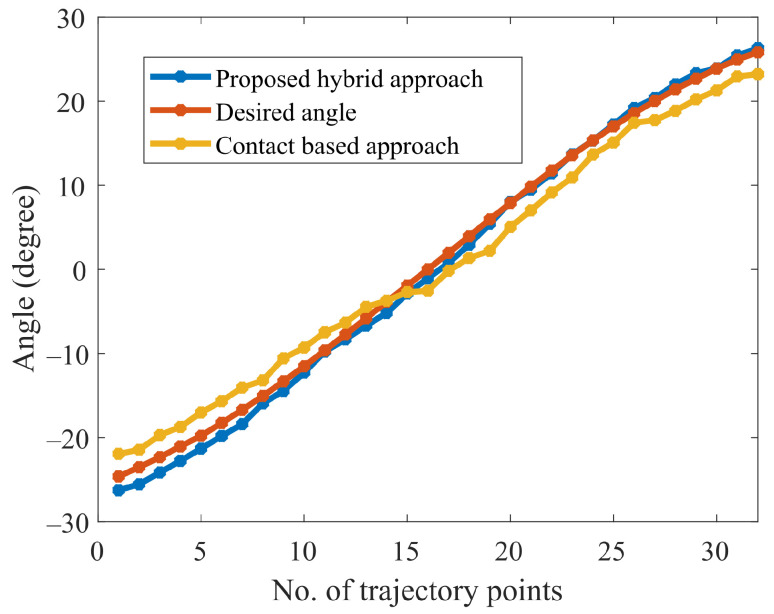
Comparison between contact-based and proposed approaches.

The explanation for the normal trajectory generation is similar to the aforementioned section. The experimental results of normal angle estimation obtained during curved surface tracking are shown in Figure 15. For comparison, a normal trajectory (the desired trajectory) is obtained from the CAD model, which is referred to as the desired trajectory in the text. The desired trajectory is used for comparing the results obtained using the proposed scheme (hybrid approach) and the recent work (contact-based approach) proposed in [31].

**Figure 16:**
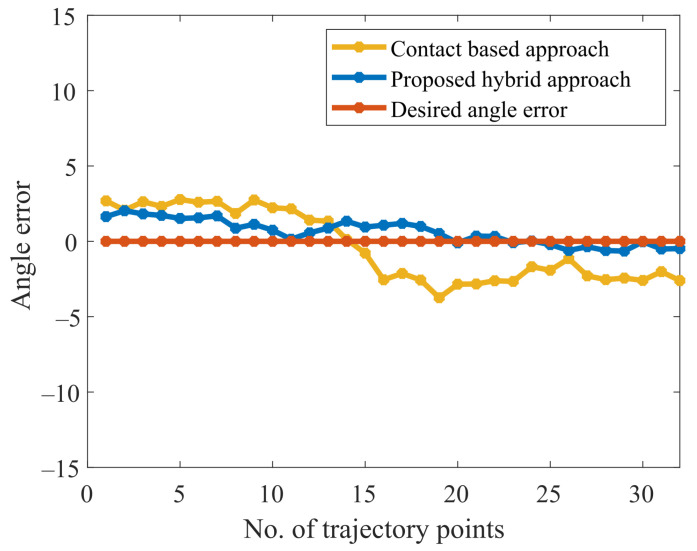
Absolute error comparison between contact-based and proposed approaches.

**Figure 17:**
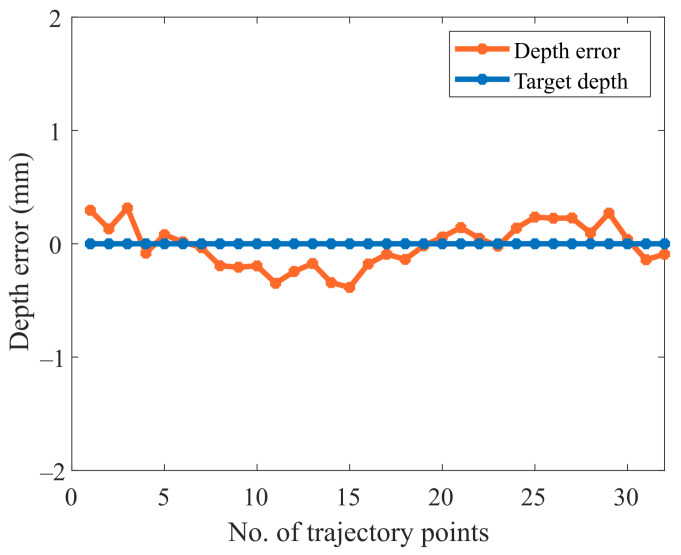
Absolute depth error while tracking the curved surface.

It is shown in Figure 15 that the obtained normal trajectory using our proposed approach, follows the desired trajectory more closely than the contact-based approach. The current discourse endeavors to provide a comparative evaluation between the experimental outcomes obtained on a curved surface and the recent work conducted by [31]. As per the findings, the mean absolute error (MAE) and standard deviation (SD) attained by Li et al. were estimated at approximately 3.2 and 3.4, whereas our proposed methodology demonstrated significantly superior performance, resulting in an MAE and SD of 0.7 and 0.5. This disparity in the respective MAE and SD values clearly highlights the potential of our proposed approach to yield more accurate and precise results in the relevant domain. On the other hand, the maximum average error obtained is 1.8 degrees, and the maximum average depth error is 0.4 mm, as shown in Figure 16 and Figure 17. It is worth noting that, in contrast to contact-based approaches, this method is feasible for most materials. However, the workpiece’s surface color and surrounding brightness significantly affect the outcomes of the experiments. To summarize the desired surface path tracking experimental findings, the system’s criteria for accuracy and real-time capability are ensured by the stereo camera and the algorithm used. The depth measurement tool mounted on the robot is able to keep vertical contact with the surface. Consequently, constant normal (perpendicular to the surface) contact on a curved surface is realized.

## 6. Conclusions

Normal trajectory generation plays a vital role in robotic-based machining operations such as laser cutting. This study proposed a novel technique to obtain a normal trajectory while tracking the contour surface path. This study deals with two challenges: desired path tracking and normal trajectory generation. Initially, a key-point approach is employed to enable the robot to reach the desired path on the curved surface using a depth measurement tool positioned on the flange of the robot. While tracking the desired path, the point cloud information is used to estimate the deviation angle from the surface normal at each point on the curved surface. Consequently, a normal trajectory is achieved on the desired path after the completion of the surface tracking. An RGB-D camera mounted on the flange of the robot is responsible for providing the point cloud data of the path. Four sparse points are developed using the center point of the point cloud data at each point on the desired path while tracking the surface. A geometric technique was employed to calculate the surface normal at any point of interest. This allows for quick and easy computation of both the position and orientation between the robot’s end-effector and the contour.

The performance of the proposed technique is analyzed on an experimental setup using a 6 DOF industrial robot. The experimental findings demonstrate that the accuracy has been improved compared to prior research documented in the literature, such as contact- and non-contact-based approaches. It is worth noting that the efficiency of the proposed scheme is highly dependent on the type and measurement behavior of the camera utilized. The findings show that this study has great potential for automating machining operations, such as laser cutting, in order to produce more precise and reliable results.

## Figures and Tables

**Table 1:** A comparative study of the proposed work and earlier research efforts.

Author	Approach	Tool	Average Error (deg)
[13]	Contact	Force/Torque sensor	5
[31]	Contact	Force/Torque sensor	2.8
[30]	Hybrid	3-laser and linear encoder	3.3
Proposed	Enhanced non-contact	3D Camera and depth tool	1.8

## Data Availability

The data presented in this study are available on request from the corresponding author. The data are not publicly available due to privacy.
